# Predicting evolutionary change at the DNA level in a natural Mimulus population

**DOI:** 10.1371/journal.pgen.1008945

**Published:** 2021-01-13

**Authors:** Patrick J. Monnahan, Jack Colicchio, Lila Fishman, Stuart J. Macdonald, John K. Kelly

**Affiliations:** 1 Department of Ecology and Evolutionary Biology, University of Kansas, Lawrence, Kansas, United States of America; 2 Division of Biological Sciences, University of Montana, Missoula, Minnesota, United States of America; 3 Department of Molecular Biosciences, University of Kansas, Lawrence, Kansas, United States of America; University of Wyoming, UNITED STATES

## Abstract

Evolution by natural selection occurs when the frequencies of genetic variants change because individuals differ in Darwinian fitness components such as survival or reproductive success. Differential fitness has been demonstrated in field studies of many organisms, but it remains unclear how well we can quantitatively predict allele frequency changes from fitness measurements. Here, we characterize natural selection on millions of Single Nucleotide Polymorphisms (SNPs) across the genome of the annual plant *Mimulus guttatus*. We use fitness estimates to calibrate population genetic models that effectively predict allele frequency changes into the next generation. Hundreds of SNPs experienced “male selection” in 2013 with one allele at each SNP elevated in frequency among successful male gametes relative to the entire population of adults. In the following generation, allele frequencies at these SNPs consistently shifted in the predicted direction. A second year of study revealed that SNPs had effects on both viability and reproductive success with pervasive trade-offs between fitness components. SNPs favored by male selection were, on average, detrimental to survival. These trade-offs (antagonistic pleiotropy and temporal fluctuations in fitness) may be essential to the long-term maintenance of alleles. Despite the challenges of measuring selection in the wild, the strong correlation between predicted and observed allele frequency changes suggests that population genetic models have a much greater role to play in forward-time prediction of evolutionary change.

## Author summary

For the last 100 years, population geneticists have been deriving equations for Δp, the change in allele frequency caused by mutation, selection, migration, and genetic drift. These equations are seldom used directly, to match a prediction for Δp to an observation of Δp. Here, we apply genomic sequencing technologies to samples from natural populations, obtaining millions of observations of Δp. We estimate natural selection on SNPs in a natural population of yellow monkeyflowers and find extensive evidence for selection through differential male success. We use SNP-specific fitness estimates to calibrate a population genetic model that predicts observed Δp into the next generation, and find that when male selection favored one nucleotide, that nucleotide increased in frequency in the next generation. Since neither observed nor predicted Δp are generally large in magnitude, we developed a method called “haplotype matching” to improve prediction accuracy. The method leverages intensive whole genome sequencing of a reference panel (187 individuals) to infer selection in thousands of field individuals sequenced at much lower coverage. This method proved essential to accurately predicting Δp in this experiment and further development may facilitate population genetic prediction in the future.

## Introduction

Natural selection is routinely strong enough to measure within wild populations. Classic experiments on conspicuous polymorphisms were the first to establish fitness differences among genotypes [1,2]. Field experiments later demonstrated selection on allozymes [3] and structural variants such as inversions [4–6], but the set of loci amenable to direct study has greatly expanded with quantitative trait locus (QTL) mapping [7]. QTLs link genotype to phenotype in way that can provide a “mechanistic” understanding of selection in terms of the processes that maintain polymorphism (e.g. antagonistic pleiotropy [4,8], frequency dependent selection [9] or gametic/zygotic fitness trade-offs [10]) and the environmental drivers of selection (e.g. differential predation [11]). In aggregate, single-locus and QTL studies have provided great insight on the contribution of major loci to the standing variance in fitness within natural populations.

Genome-wide surveys of natural populations deliver a comprehensive view of selection. An important question is how many loci across the genome experience selection in a typical generation. Sequencing of natural populations sampled through time suggests that the strong selection documented in single locus studies can occur at hundreds of polymorphisms simultaneously [12,13]. In *Drosophila melanogaster*, large amplitude fluctuations in allele frequency occur seasonally and can be directly related to weather conditions [14]. The magnitude and consistency of changes, as well as the environmental correlation, clearly imply that selection (and not genetic drift) is causal. The temporal sampling method employed for *D*. *melanogaster* should be applied to other systems [15], but some questions require individual-level data. For instance, are fitness differences caused mainly by differences in viability or fertility or mating success? Experiments predicting individual fitness from individual genomes have been conducted in a variety of organisms using both “common gardens,” where sequenced individuals are transplanted into natural settings [16–19], as well as by monitoring of native individuals *in situ* [20–22]. These studies yield varying results on the importance of different selection components, but in aggregate, suggest that selection is a pervasive force on ecological time scales.

Here, we measure genome-wide selection and allele frequency change in a field study of *Mimulus guttatus*; a plant in which all of the different methods described above have been applied to a single natural population in central Oregon, USA (Iron Mountain, hereafter IM). We have demonstrated strong fitness effects of segregating inversions by genotyping IM plants that were also scored for fecundity [23,24]. Transplant experiments using plants that differ only at QTL for ecologically important traits have confirmed that conflicting selection pressures are key to the maintenance of variation [25,26]. QTL alleles that increase plant size at reproduction tend to delay flowering, which generates antagonistic pleiotropy between survival and fecundity. These single-locus experiments (QTLs and inversions) have been corroborated by Genome Wide Association (GWA) of traits and fitness components in IM [18]. ‘Big/slow’ alleles that delay progression to flowering, but increase flower size, segregate at many loci across the genome. They tend to be less frequent than their ‘small/fast’ alternatives within IM [18,27], which is consistent with many years of field monitoring indicating that viability selection generally favors small/fast alleles [25,26,28]. However, the GWA also demonstrated temporal fluctuation in the net balance of fitness components [18] suggesting that year-to-year changes in water availability are key to the maintenance of variation.

The focus of this paper is prediction: Can we characterize selection at the SNP level accurately enough to predict allele frequency change into the next generation? Prospective (forward-time) prediction of evolutionary change from measurements of selection is a primary goal of quantitative genetics [29–33], but has long been considered beyond the scope of population genetics [34]. In quantitative genetics, estimates of phenotypic selection (differentials or gradients) can be combined with estimates of inheritance (heritability or genetic (co)variance) to predict $\Delta \bar{z}$, the change in mean phenotype [35,36]. Prediction accuracy can be improved by directly relating the loci affecting a trait to fitness, using either the secondary theorem of selection [37,38] or via genomic selection methods [39]. The scope of quantitative genetics is broad, but its enduring relevance to both agriculture [40,41] and evolutionary biology [30] rests largely on its capacity for prospective prediction. It is an open question whether selection on SNPs strong enough to predict Δ*p*, the change in allele frequency, in a manner analogous to $\Delta \bar{z}$.

To estimate selection on SNPs, we sequenced reduced representation [42] DNA libraries from 1936 experimental plants (field individuals and progeny). We called variants within reads and aligned them to 187 full genome sequences previously obtained from the IM population [18]. This alignment is the basis for the “haplotype matching” technique of genotype inference. We then apply haplotype matching to derive genotype probabilities for SNPs within 15,360 genic regions of experimental plants. These likelihoods are inputs to the selection component models that predict allele frequency change [20,43]. We estimate male selection by synthesizing maternal and progeny data to infer the (unseen) male siring fitness component. We show that male selection in 2013 predicts observed changes in allele frequency into the next generation; the latter estimated from a distinct sampling of plants in 2014. We then describe haplotype matching in detail and provide a proof-of-concept application to data from the *Drosophila* Synthetic Population Resource (DSPR) [44], where haplotype inheritance is known. Finally, we consider the genomic scale of natural selection by integrating field estimates from 2014 with those obtained from the previous generation.

## Results and discussion

**Fig 1:**
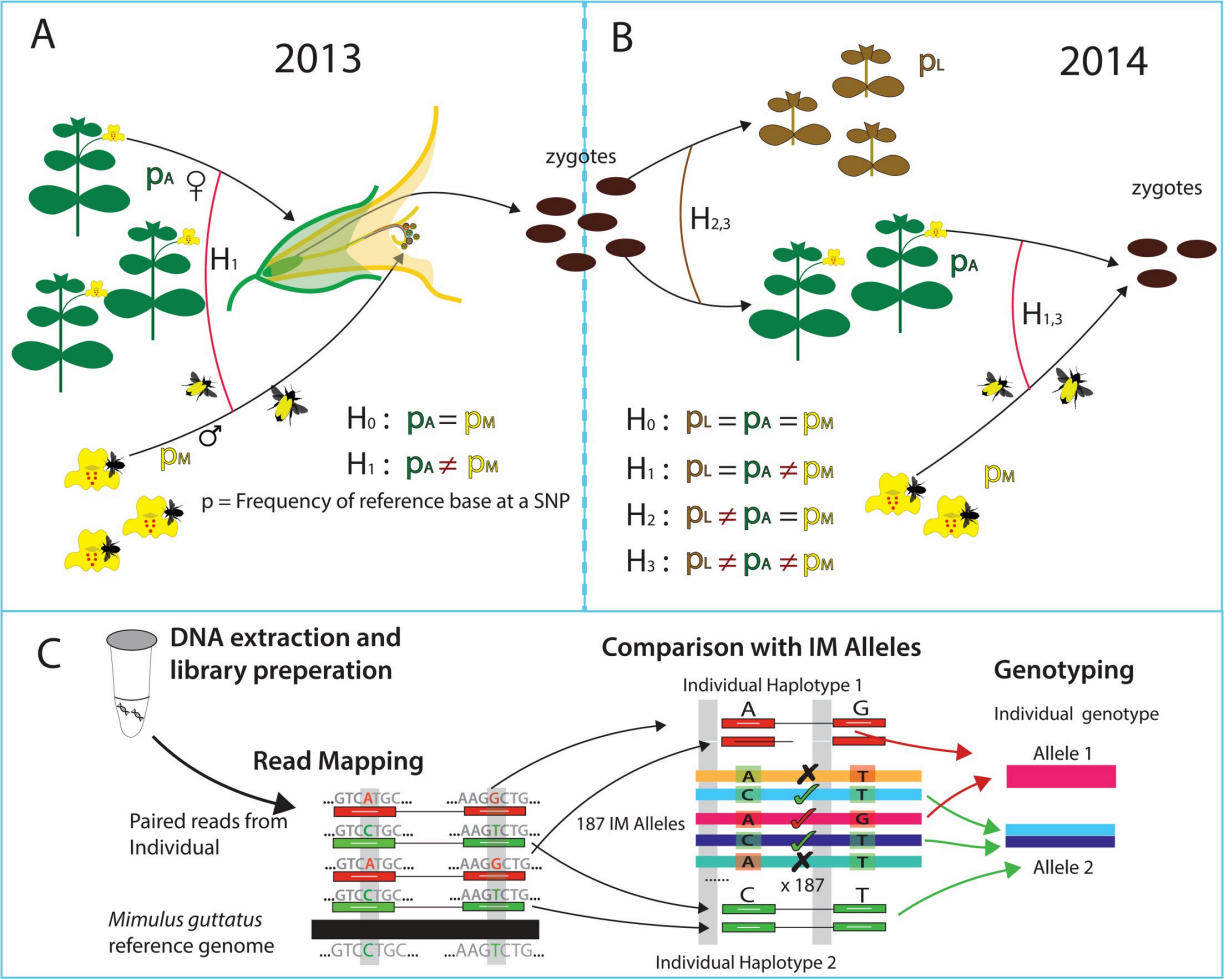
The parameters of alternative selection models are depicted for the (A) 2013 and (B) 2014 data. Hypothesis tests are expressed in terms of parameter constraints where p indicates reference base frequency: p_A_ for reproductive adults, p_M_ for successful male gametes, and p_L_ plants for plants that fail to reproduce. H_0_ is the full neutral model. Male selection is tested by contrast of H_1_ to H_0_ in 2013 and H_3_ to H_1_ in 2014. Viability selection is tested by contrast of H_3_ to H_2_. (C) After DNA sequencing, read-pairs are mapped to the *M*. *guttatus* reference genome. The haplotype matching method (read-pairs to genic-haplotypes) is illustrated for a simple case with read-pairs mapping to single location. Read-pairs impose a probabilistic ‘process of elimination’ on reference line sequences as putative ancestors: √ indicates consistency and “X” inconsistency.

*Mimulus guttatus* (syn. *Erythranthe guttata*) is a hermaphroditic species that can experience selection prior to flowering, via differential viability, and subsequent to flowering through both male and female function. In the first year of our study (Fig 1A: 2013), we sampled plants that successfully flowered (adults) and genotyped them using MSG-RADseq [42] reduced representation sequencing. We also grew and genotyped a random collection of progeny from each adult. Given the maternal genotype, we can statistically identify her allelic contribution to offspring and distinguish allele frequency among all adults (*p*_*A*_) from that in the population of *successful* male gametes (*p*_*M*_). The *p*_*A*_/*p*_*M*_ test evaluates whether these frequencies are different and thus identifies selection through differential male success. “Male selection” integrates a number of distinct selective mechanisms [20] including simple differences in fecundity (which may be equivalent between male and female function), sexual selection through differential siring [45] and pollen competition [46].

To test the predicted changes caused by male selection in 2013, we sampled plants from the next generation (Fig 1B: 2014). In 2014, we genotyped three distinct cohorts: individuals that germinated but failed to reproduce (allele frequency *p*_*L*_), individuals that successfully flowered and produced fruit (allele frequency *p*_*A*_), and a random sample of progeny from reproductive individuals (used to estimate *p*_*M*_). We estimated allele frequencies using a two-stage genotyping strategy (haplotype matching) described and tested in the next section (Fig 1C). We then performed statistical contrasts between cohorts, asking whether allele frequency differs using likelihood-based selection component models [43,47–49] generalized to accommodate uncertain genotype calls [20]. Selection is indicated when a model that allows allele frequencies to differ between cohorts, e.g. *p*_*A*_≠*p*_*M*_, has a much higher likelihood than a constrained model, e.g. *p*_*A*_ = *p*_*M*_ (see Materials and methods section D).

### Male selection in 2013 predicts evolution into 2014

**Fig 2:**
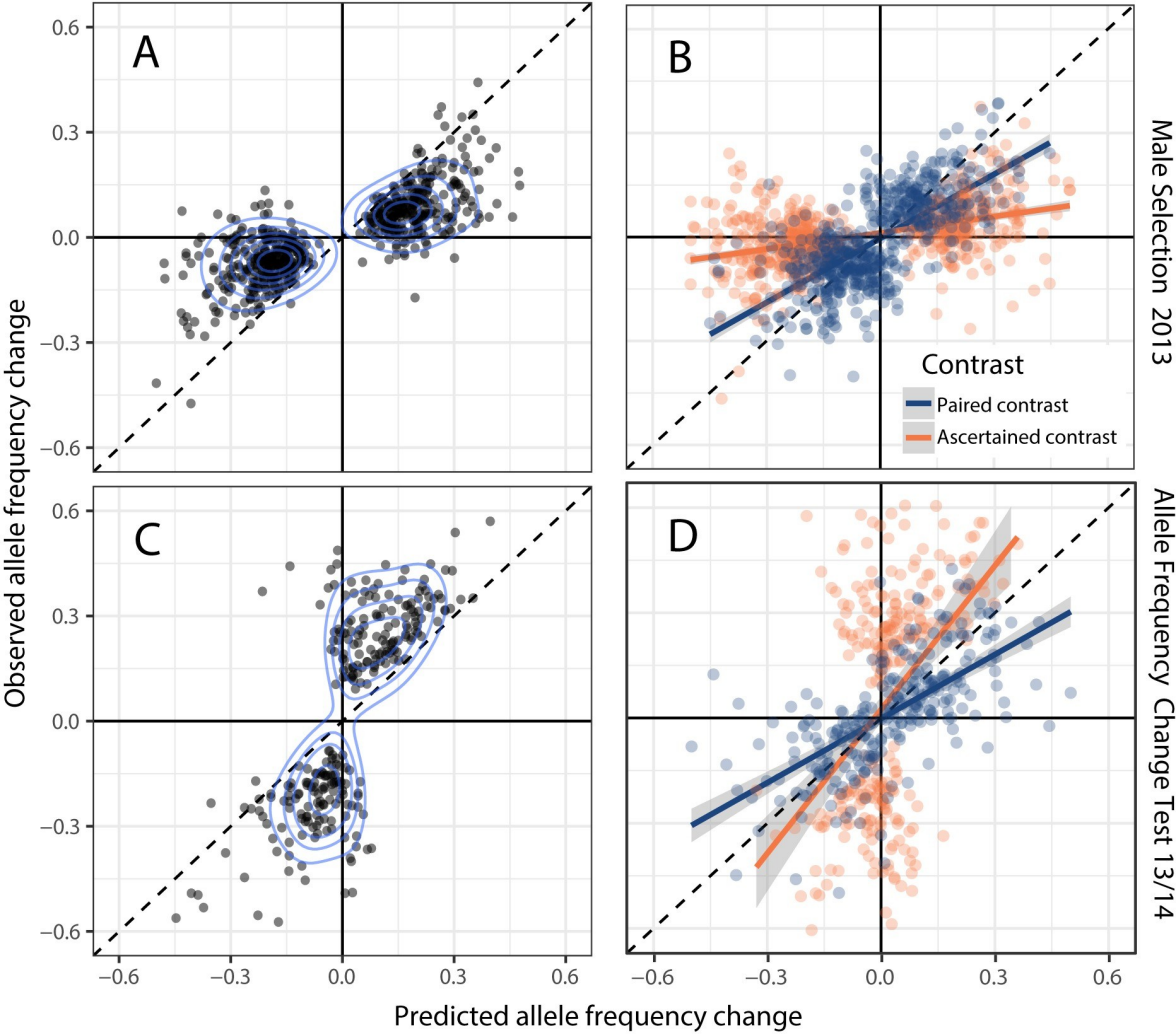
**The observed allele frequency change (2013 adults to 2014 zygotes) is compared to predicted with SNPs chosen based on (A) significance for male selection in 2013 (n = 587) or (C) significance for the Change in Allele Frequency test (n = 274).** Results are reported for all gene sets with a SNP with p < 10^−5^. Contours indicate the density of points in panels A,C. For cross-validation (B, D), we split the data into two halves and performed model fits on each half. We chose an equivalent number of SNPs to the corresponding un-partitioned analyses with n = 587 in (B) to match (A) and n = 274 in (D) to match (C). For SNPs selected based on male selection (B), the “Ascertained” contrast is based on the predicted Δp from the significant test (orange points) while the “Paired” contrast is based on the predicted Δp from the other half of the data (blue points). (D) In the cross-validation for allele frequency change significant tests, the ascertained (orange) is the observed Δp from the significant test and predicted Δp from the other data half. Assignment is reversed because the allele frequency change test is based on the observed Δp.

We evaluated prediction accuracy by ascertaining SNPs in two ways, first those with the strongest evidence for selection and then those with the strongest evidence for change. We tested 1,523,410 SNPs within genic regions (filters described in Materials and methods section B). For male selection in 2013, 1337 SNPs were genomewide significant with *p*_*A*_/*p*_*M*_ test p-values less than the Bonferroni threshold (α = 0.05/1523410), although many of these SNPs are in very strong linkage disequilibria (within the same gene). After thinning to the single most significant test per gene, 112 remained. Given that Bonferroni is excessively conservative, we conducted subsequent analyses accepting SNPs (at most one per gene set) with p < 10^−5^ (587 SNPs in Fig 2A). For *p*_*A*_/*p*_*M*_ in 2013, the 10^−5^ cut-off corresponds to a false discovery rate of 0.002 using the Benjamini-Hochberg method [50]. Fig 2A contrasts the predicted change, Δ*p* = (*p*_*M*_−*p*_*A*_)/2, to the apparent Δ*p* from 2013 adults to 2014 zygotes. To identify the SNP with the greatest evidence for change, we consider the data from both years simultaneously in the Allele Frequency Change Test of Fig 2C. We first fit a model where *p*_*A*_ in 2013 is constrained to equal *p*_*Z*_, the allele frequency in zygotes of 2014 (null hypothesis). We contrast that likelihood to a more general model where *p*_*Z*_ of 2014 is allowed to differ from *p*_*A*_ in 2013, its value determined entirely by data from 2014. Rejecting *p*_*A*13_ = *p*_*Z*14_ indicates allele frequency change into the next generation. Applying this test, we find that 274 gene sets have at least one SNP with p < 10^−5^ (Fig 2C).

We obtain strongly positive relationships between predicted and observed allele frequency change from both male selection and allele frequency change tests (Fig 2A: r = 0.79; Fig 2C: r = 0.76; p<0.0001 for both). Each relationship deviates from 1:1 (the naïve expectation with unbiased prediction) with the slope for male selection SNPs less than 1 (A: 0.40) and the slope for allele frequency change SNPs greater than 1 (C: 1.57). The evident positive associations between observed and predicted Δ*p* are very encouraging, but these relationships require careful statistical scrutiny. The data (and thus estimates) from 2013 and 2014 are statistically independent, but the x- and y-axis Δ*p* values in Fig 2A and 2C share a parameter (*p*_*A*_ in 2013) that contributes negatively to the values on each axis. As a consequence, estimation error in *p*_*A*_ will generate a positive covariance between observed and predicted *apart from that generated by correct prediction*. Ascertainment is second factor. Choosing the most significant SNP for male selection in 2013 will select for those with exaggerated estimates of (*p*_*M*_−*p*_*A*_). When male selection favors the reference base, the most significant tests will have positive estimation error added to the true positive value of (*p*_*M*_−*p*_*A*_), and the opposite is true for SNPs where the alternative base is favored [51]. The so called “winner’s curse” [52,53] will thus reduce the regression slope relative to 1 in Fig 2A because the allele frequency in 2014 zygotes is unaffected by estimation error in the previous generation. Ascertainment tends to exaggerate the y-axis variable for the allele frequency change test, inflating the slope relative to one. The regression slopes in Fig 2A and 2C (observed onto predicted) deviate from 1:1 as predicted by this ascertainment effect.

We conducted two analyses that establish genuine prediction of Δ*p* in the face of these errors and biases. First, we used ‘cross-validation’ by splitting the 2013 experiment into odd numbered and even numbered families, respectively. We then performed model fits on each half separately, generating two distinct pairs of observed and predicted Δ*p* for each SNP. We then matched the “odd” predicted Δ*p* to the “even” observed Δ*p*, and vice versa (two distinct contrasts for each SNP). The contrasts are not equivalent because the test ascertained as significant will reside (usually) in only one data half. We denote the “Ascertained contrast” as the one with the significant test Δ*p* (say Odd) matched to the observed Δ*p* from the other data half (even). The remaining data from this SNP (predicted from even, observed from odd in this example) is the “Paired contrast.” With cross validation, there is no correlation in the absence of prediction (confirmed by simulation in S1D Appendix).

The split data produce strong positive relationships between observed and predicted Δ*p* for both Ascertained and Paired contrasts (Fig 2B and 2D) despite the reduction in power caused by halving the data. For male selection (Fig 2B), correlations between predicted and observed would be zero for both Paired and Ascertained if SNPs were neutral (or prediction unrelated to response at non-neutral SNPs). In fact, both correlations are highly significant (p < 0.00001 for each in Fig 2B). Importantly, the regression slope is greater for the Paired contrasts (0.62) than the Ascertained contrasts (0.16). This is expected because the magnitude of predicted Δ*p* values is substantially greater in Ascertained relative to Paired contrasts. The exaggeration of predicted Δ*p* inherent to the former group (winner’s curse) reduces the slope. Finally, we note that the predicted Δ*p* is strongly correlated between data halves (r = 0.86, n = 587, p < 0.00001). No correlation is expected under neutrality.

Cross-validation for the Allele Frequency Change test required subdivision of data from both years. We split the 2014 data into even and odd families and (arbitrarily) combined 2013-odd with 2014-odd. Then, as previously, we fit models (here the Allele Frequency Change test) to each data half for each SNP and identified the most significant test per gene. As previously, both Ascertained and Paired contrast sets produce strongly positive correlations between observed and predicted Δ*p* values (p < 0.00001 for each in Fig 2D). Here, the regression slope is lower with Paired (0.61) than Ascertained SNPs (1.29). This change in pattern regarding the slopes between in Fig 2B and 2D is predicted given the nature of ascertainment for the allele frequency change test. Here, the observed Δ*p* will be inflated relative to the truth for Ascertained but not for Paired contrasts.

As a complement to cross-validation, we developed a full genome simulation program to generate data under the condition that prediction is ineffective (no true relationship between observed and expected). This simulator (S1D Appendix) produces read-pair data equivalent in structure and amount to the real data. To this output, we can apply the full bioinformatic pipeline applied to the actual data. The simulated data reiterates estimation error and is subject to the same ascertainment biases as the actual data, but without allele frequency change. The latter is assured because we sample genotypes randomly (fitness is equal for all genotypes).

We first applied the selection component models to simulation outputs to confirm our methodology for calling test p-values. When there is no selection, we find that the sampling distribution of Likelihood Ratio Test statistic follows the chi-square density, consistent with the asymptotic normal theory for likelihood testing (S1D Appendix). This is how we calculated p-values on tests with the actual data. Second, we confirmed that the cross-validation method eliminates the spurious association between predicted and observed Δ*p* (null hypothesis for Fig 2B and 2D). Finally, the simulations confirm that a positive association between observed and predicted change is generated by estimation error in the un-partitioned data (Fig 2A and 2C). However, the covariance between observed and predicted is much greater for the real data than for the simulated data (0.020 vs 0.012 for male selection, 0.033 vs 0.012 for the Allele Frequency Change test). Thus, the magnitude (if not simply the direction) of the covariance in Fig 2A and 2C is indicative of effective prediction. In summary, the simulation and cross-validation procedures provide strong support that prediction is genuine.

### The haplotype matching method

We derived SNP allele frequency estimates in two stages (Fig 1C). In the first, we map read-pairs to the set of ‘genic haplotypes’ present in IM. Sequence variation is very high in *M*. *guttatus* [54] and it is difficult to effectively call variants outside genic regions. We thus established “gene sets” as loci. A set is either a single gene or a collection of closely linked (within 100bp) and/or overlapping genes (S1 Table). The genic haplotypes are the sequences for this locus among the reference panel genomes (detailed procedures in S1B Appendix). With 187 distinct haplotypes, there are 17,578 distinct genic-genotypes. However, most gene sets have fewer than 187 because some IM lines are identical within a gene set (the median number of distinct haplotypes is 100 per gene, S1 Table).

We treat the genic haplotypes as the sequences present in the natural population (Fig 1C). Let *U*_[*plantID*],*i*,*j*_ denote the likelihood for the full collection of read-pairs from a plant given that its diploid genic-genotype is [*i*,*j*], where *i* and *j* index genic haplotypes. For an outbred plant,

$$U_{[plantID],i,j}=\prod_{r=1}^{RP}\left(\frac{\epsilon^{h_{r,i}}}{2}+\frac{\epsilon^{h_{r,j}}}{2}\right)$$ (1)

where RP is the number of read-pairs mapped in this gene set, *h*_*r*,*i*_ is the number of sequence mismatches between read-pair *r* and genic haplotype *i*, and *ϵ* is the mismatch probability. *ϵ* aggregates the various events (sequencing error, alignment error, etc) that could create an apparent sequence difference even if the read-pair and haplotype are the same. *U* relates the RADseq data collected from field plants to the tests for selection.

A potential difficulty with haplotype matching is that the sequence of a field plant may not match any of our genic haplotypes owing to recombination. This will reduce our power to detect selection, potentially generating false negatives but not false positives [51]. It is straightforward to test whether individual read-pairs are consistent with the genic haplotypes. Across the 99 million read-pairs in the final RADseq dataset (both years), the median number of SNPs per read-pair is 6. About 20% of read-pairs overlap 10 or more SNPs (S2 Table, S1 Fig). Across all read-pairs, less than 0.2% failed to perfectly match at least one genic haplotype. Of course, the full collection of read-pairs from a plant can still be inconsistent with any pair of genic haplotypes (even if all individual read-pairs map perfectly). This occurs, but very infrequently, and in these cases, the genotype is treated as unknown.

**Fig 3:**
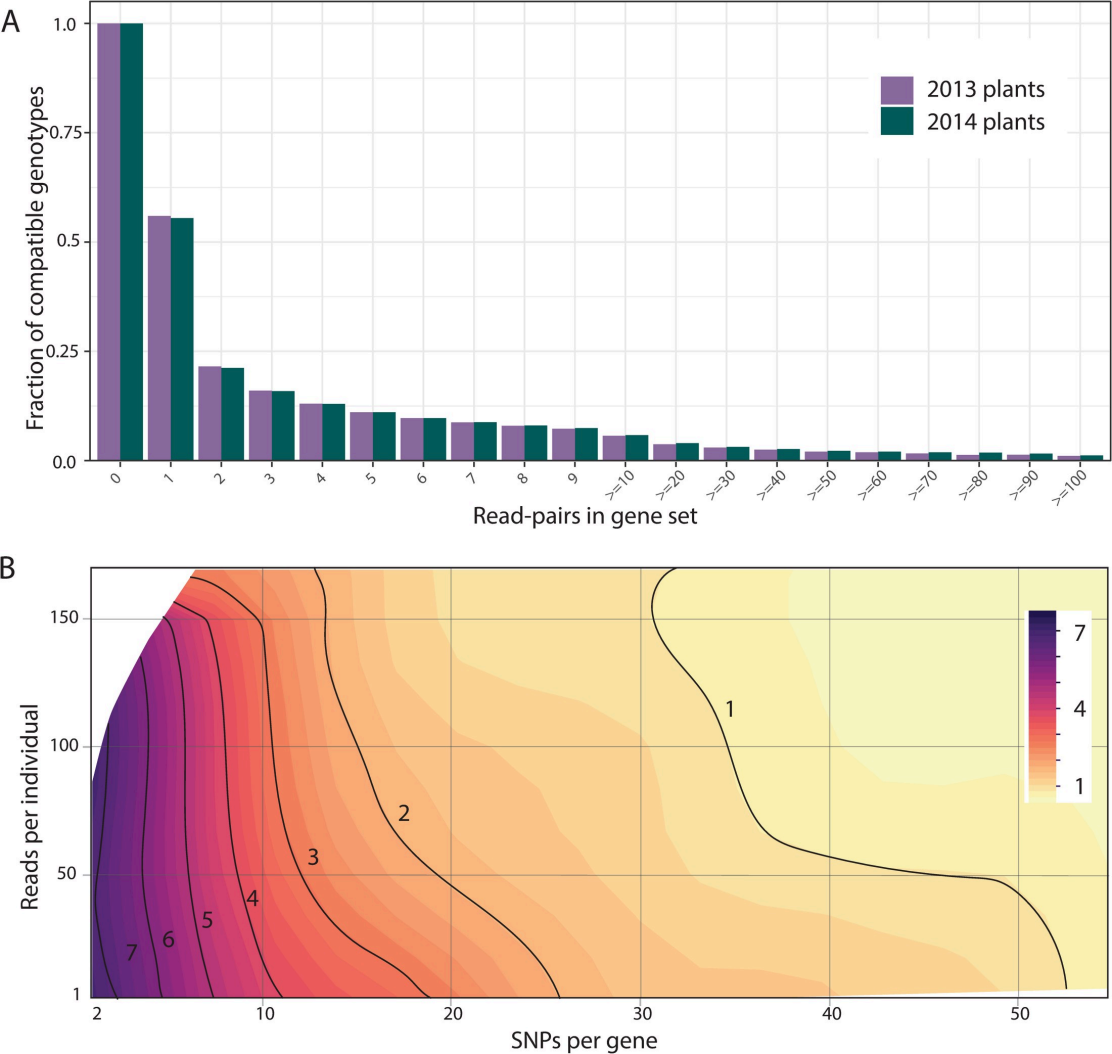
**Testing haplotype matching: (A) In Mimulus, the precision of estimation is depicted as a function of the amount of data per plant.** Compatible means that the likelihood for a genic-genotype is within 50% of the most likely genotype. (B) In Drosophila, the number of ancestors (indicated by contours and color) matching the genotype of a particular RIL is depicted as a function of amount of data (reads) and the number of SNPs in the gene set.

Given consistency, the question becomes how precisely low-level sequencing can identify the genotype of field plants. As expected, the number of possible genic genotypes for a plant declines as the number of read-pairs mapped to a gene set increases (Fig 3A). With low but reasonable coverage (10–20 read-pairs over an entire gene), the collection of compatible genic-genotypes is greatly reduced (on average to ≈5% of the total). Oftentimes, we identify one parental genic-haplotype definitively, but the other is consistent with multiple sequences from the reference set (illustrated by Fig 1C). The aggregation of evidence across numerous read-pair loci (mapping to different parts of gene) is usually needed to identify specific genic-haplotypes. While zeroing in on 5% of diploid genic-genotypes is still hundreds of possibilities, these possibilities often strongly “agree” about the genotype at particular SNPs when nearly all genic-genotypes have the same bases at that SNP. SNP specific inference can be quite strong even with moderate coverage. Plants with low sequencing coverage often have few or no read-pairs, particularly in smaller gene sets. In isolation, inference for such plants would be weak. Here, inference can become much stronger with information from relatives (the maternal plant, siblings, or offspring). Importantly, we never truncate probabilities to produce “hard calls” for SNPs. Uncertainty is propagated through the entire analysis and thus properly integrated in testing. The selection analyses cycle through all SNPs within a gene set, considering each as a potential effector of fitness.

### A test of haplotype matching with data from *Drosophila melanogaster*

We used haplotype matching to estimate allele frequencies for the tests in Fig 2. With the *Mimulus* data, we do not know the true genic-genotype of field plants and thus cannot compare inferred to known. For this reason, we applied our pipeline to a *Drosophila melanogaster* population where genic-genotypes are known with high confidence. The *Drosophila* Synthetic Population Resource (DSPR) consists of two multiparental, advanced generation intercross Recombinant Inbred Line (RIL) populations, each initiated from eight inbred founder strains [44,55]. The fully sequenced founder strains represent the reference panel in the current context. The RILs (comparable to Mimulus field plants) were genotyped and we know the founder strain that contributed the allele at each gene of each RIL. Some regions in some RILs are not genotyped with certainty, but we exclude these from our analyses.

We collected MSG-RADseq data on 60 of the RILs from DSPR using the same methods as for Mimulus, except that the Drosophila sequences are 94bp single end reads instead of the PE100. We processed the *D*. *melanogaster* reference genome into ‘gene sets’ and then implemented the same Mimulus pipeline for read mapping, SNP calling and haplotype matching. The great majority of *D*. *melanogaster* reads overlap 3 or fewer SNPs and are thus less informative than the Mimulus read-pairs (S1 Fig). Finally, we compared the inferred genotype to the “known” ancestry of each RIL as a test of the method.

This exercise confirms the validity of the haplotype matching, but also its limitations. The ancestral line (or lines) deemed most likely by haplotype matching includes the “correct” line ≈99.5% of the time. We assigned the ancestral genotype as “known” if the posterior probability was greater than 0.99 [44,55] and thus a small rate of mismatch (less than 1%) is expected even if haplotype matching is perfect. The 99.5% obtained by haplotype matching of MSG data is thus actually close to the theoretical upper limit for accuracy. However, while haplotype matching is accurate, it is not always precise. Oftentimes, the method predicts that numerous genic-genotypes are equally likely. Inference to the specific correct ancestor increases in a predictable fashion with the number of SNPs per gene set and number of reads scored for that line (Fig 3B).

### The scale of genome-wide selection

**Fig 4:**
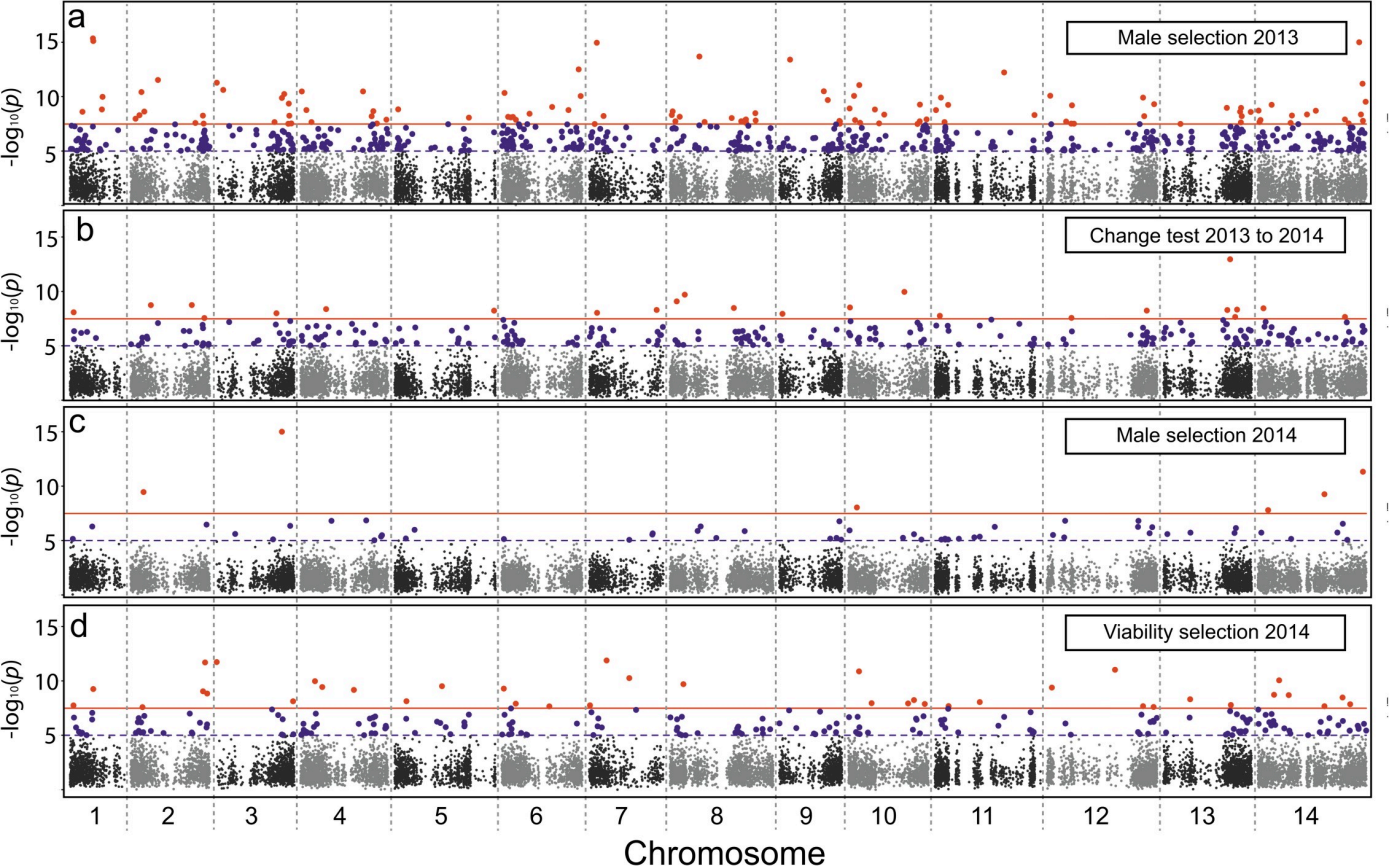
Manhattan plots, with a single test reported per gene, for (a) Male selection 2013, (b) Allele frequency change 2013–2014, (c) Male selection 2014, and (d) Viability selection 2014. The orange line is the Bonferroni threshold, purple is p = 10^−5^.

**Fig 5:**
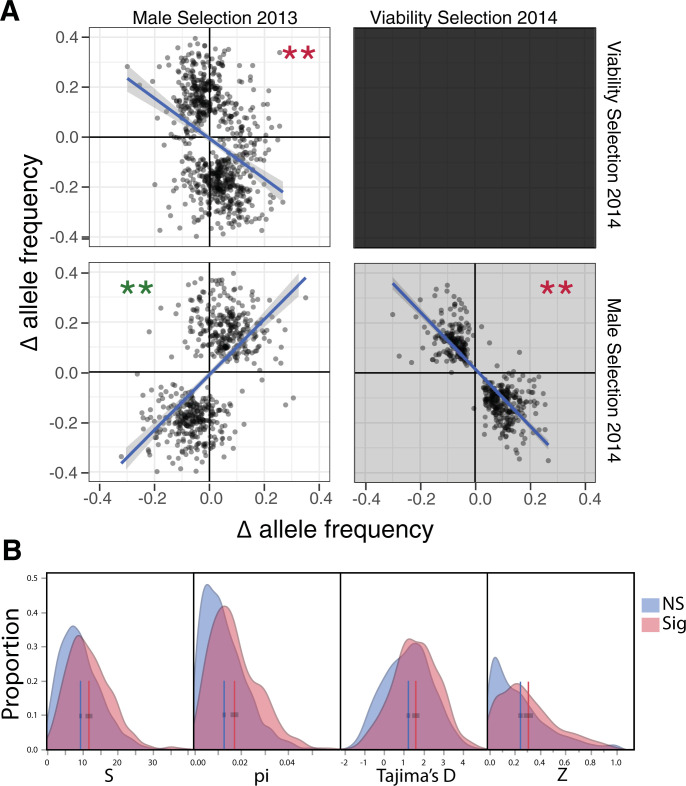
**(A) Pairwise contrasts between predicted changes owing to male selection in 2013, viability selection in 2014, and male selection in 2014.** A single SNP per gene is reported (the most significant) if p < 10^−5^. The SNPs ascertained for contrasts are distinct from those for tests in isolation (Fig 4), although these SNP sets are overlapping (see S5 and S9 Tables). (B) Density plots for molecular test statistics are depicted for 587 selected-SNP windows and 2751 control windows. Vertical lines are means. The number of polymorphisms (S), nucleotide diversity (pi), and Tajima’s D are based on the flanking DNA (100 bp on each side, but not including the focal SNP). Z focal is Z_ns_ calculated by contrasting the focal SNP to all flanking SNPs.

A principal motivation for genomic Selection Component Analyses is to determine how much selection is occurring across the genome in a typical generation. We found abundant evidence for selection in 2013 (Fig 2), and also when estimating selection components from the plants in 2014 (Figs 4 and 5). For Fig 2, we used the 2014 data simply to estimate the observed Δ*p* from 2013–2014, but the experimental design allows a more detailed dissection of fitness variation within 2014 (Fig 1B). Viability selection estimated from the difference between *p*_*A*_ and *p*_*L*_ was abundant in 2014 with 226 genes having at least one SNP tests with p < 10^−5^ (Fig 4D). Genes were significantly more likely to exhibit viability selection in 2014 if under male selection in 2013 ($X_{\left[1\right]}^{2}$ = 21.8, p<10^−5^). Male selection was considerably weaker in 2014 than 2013 with only 59 genes having a SNP with p < 10^−5^ for the *p*_*A*_/*p*_*M*_ test (Fig 4C). As with viability selection, there was a strong tendency for genes significant in 2013 to also be significant for male selection in 2014 ($X_{\left[1\right]}^{2}$ = 53.4, p<10^−5^). The genomic locations of the ‘best-per-gene’ results for all four tests (2013 and 2014) are depicted in Fig 4 and S5 Table. The broad distribution of significant tests across chromosomes suggests extensive selection in IM (Fig 4).

The genomic extent of selection is a fundamental question in evolutionary biology. The concern that selection acting simultaneously at many loci generates excessive genetic load, i.e. the cost of selection [56,57], was a major impetus for the development of the neutral theory of molecular evolution [34,58]. In this context, the sheer number of significant tests in Fig 4 seems surprising. Considering the estimated Δ*p* across a full generation (adults of 2013 to adults of 2014) at the 587 SNPs that were significant male selection in 2013, the median increase of the favored allele was 0.045. This estimate is inflated by ascertainment, but still useful to consider given that a selection coefficient of at least 0.16 is required to generate Δ*p* = 0.045. If loci combine multiplicatively, selection at many loci imposes an enormous variance in fitness on the population (S1G Appendix). The total variance in fitness of a real population is limited by reproductive constraints [34] and the genetic component of that variance will be only part of the total. The cost of selection is alleviated if fitness effects do not combine multiplicatively across loci (as assumed in S1G Appendix and also in much of population genetic theory, e.g. [59]). If selection acts by truncation or by other mappings from genotype to fitness, a great deal more allele frequency change can occur given the total variance in fitness [60–62].

An alternative way to alleviate the cost of selection is via linkage disequilibria (LD). There would be no difficulty with change at many loci if all positively selected alleles were in LD. Then, a single ‘selective event’ simultaneously changes allele frequency at many correlated SNPs, as occurs routinely with inversion polymorphisms. LD estimated from our reference sequences provide no support for this explanation. Pairwise LD among the 587 male selection SNPs (Figs 2A and 4A) are generally very low (S8 Table). However, our reference line genomes are 6–12 generations of self-fertilization removed from the outbred field plants that are experiencing selection [18]. Recombination in the first few generations of line formation would not have reduced LD on small genomic scales (at least not much), but it could have erased more diffuse LD among less closely linked loci. This is relevant as strong directional selection on a quantitative trait can generate diffuse LD even among unlinked loci, a phenomenon known as the ‘Bulmer effect’ [63]. In this context, the quantitative trait is lifetime reproductive success, and the way that loci combine to determine its value remains an outstanding question in evolutionary biology.

### The maintenance of polymorphism

Given many loci under selection, the question becomes how both alleles can persist. The contrast of results from 2013 and 2014 immediately suggests antagonistic pleiotropy and temporal fluctuations in fitness as potential mechanisms. Across SNPs, we see relative consistency in male selection across years, but a clear trade-off between male selection in 2013 and viability in 2014 (Fig 5A). To compare different components of selection, we selected the SNP within each gene set with the highest aggregate evidence for selection using Fisher’s combined probability statistic ([64], S1E Appendix). Alleles favored by male selection in 2013 were also favored by male selection in 2014 (r = 0.57 between the predicted Δp values from each component (Fig 5A), n = 555, p<10^−48^). Male selection favored the same allele in both years in 82% of 555 genes having a SNP with a combined p-value < 10^−5^ (S9A Table). In contrast, alleles favored by male selection in 2013 were usually disfavored by viability selection in 2014 (n = 725, r = -0.34, p<10^−20^), with 70% of SNPs exhibiting conflicting directions of selection (S9B Table). As expected from these results, there is also a negative correlation between male selection and viability within 2014 (r = -0.83; S9C Table), but testing is complicated for this contrast because the two tests share a common parameter and is thus subject to biases discussed previously.

Year-to-year changes in the pattern of selection, demonstrated in many previous experiments on IM [18,23–26,28], are also evident in this study. For example, male selection was much stronger in 2013 than 2014 (Fig 4). Temporally fluctuating fitness is often disregarded as a mechanism of balancing selection because simple models suggest protected polymorphism is unlikely [65,66]. However, subsequent theoretical studies (e.g. [60,67,68]) have shown that fluctuating selection can greatly elevate the genetic variance owing to factors like an autocorrelation of conditions between generations and/or the occasionally input of novel mutations. In the IM population of *M*. *guttatus*, two potential balancing mechanisms (temporal variation and antagonistic pleiotropy between survival and fecundity) act simultaneously on the same polymorphisms.

Balancing selection at a locus can leave an imprint in local patterns of sequence variation. If selection preserves alternative alleles for long periods, these alleles will accumulate mutations at closely linked sites elevating variation. Here, we tested this prediction by calculating molecular summary statistics within 200bp windows around the 587 SNPs identified for male selection in 2013 (Figs 2A and 4A) using our reference genomes as a population sample of sequences (S1F Appendix provides a full description of these calculations). We contrast the selected-SNP windows to a “control set” of windows around SNPs with non-significant tests. Fig 5B illustrates that the selected-SNP windows are significantly elevated relative to controls in terms of amount of nucleotide variation (S and Pi), the intermediacy of allele frequencies (Tajima’s D [69]), and strength of association between alleles (Z_ns_ [70]). The differences in means are highly significant (F > 35, p < 0.0001 for each statistic) and in the direction predicted by balancing selection on the selected-SNPs. These same tendencies obtain when considering windows around the selected SNPs from 2014 (S1F Appendix).

The elevated sequence variation around selected-SNPs (Fig 5B) is intriguing but preliminary. There may be an ascertainment bias if it is simply easier to detect selection at loci with more intermediate allele frequencies and strong haplotype structure (elevated Tajima’s D and Z_ns_). Trans-species polymorphism provides a more direct demonstration of long-term balancing selection. For example, many of the seasonally-fluctuating SNPs in *Drosophila melanogaster* are also polymorphic in the closely related species, *D*. *simulans*, suggesting that balancing selection has been acting since the common ancestor of this species [12]. We cannot evaluate this prediction here because we do not have polymorphism data for a closely related species that does not inter-breed with *M*. *guttatus*. However, the fact that divergent lineages within the *M*. *guttatus* species complex (e.g. *M*. *nasutus*, *M*. *decorus*, and perennial populations of *M*. *guttatus*) contribute alleles to the IM population [54,71] may provide a source of selectively relevant variation. Future studies should examine whether SNPs under selection within IM also exhibit adaptive differentiation among populations or lineages within the species complex.

## Conclusions

Regarding the genomic scale of selection, recent studies in fully pedigreed populations of birds and mammals have clearly shown substantial allele frequency change through time [22,72,73]. The challenge has been to attribute changes to natural selection as opposed to genetic drift [22,72]. In the present study, the sampled population (n) is about 1000 individuals from each year, which is orders of magnitude smaller than the number of reproductive individuals within the population (N) each generation [54]. The null hypothesis in our tests for selection is “experiment-level” drift (differences in allele frequency between cohorts is caused by the finite numbers of parents and offspring). Population drift is necessarily much weaker than experiment-level drift because n << N. Significant tests thus clearly indicate selection, albeit with the caution that negative results (non-significant tests) do not imply that SNPs are evolving neutrally. Perhaps the simplest confirmation of natural selection as the principle driver of Δ*p* is the contrast between years (Fig 5A). If apparent changes were caused by sampling and/or estimation error, the direction of change would not be correlated between the independent datasets of 2013 and 2014 plants.

Figs 4 and 5 indicate strong, but often antagonistic, selection on hundreds of genes. The apparent trade-off between fitness components, as well as the correlations between allele frequency and direction of Δ*p*, extend and corroborate previous experiments on this population. Fig 5A provides further evidence that montane, annual populations of *M*. *guttatus* exhibit a life-history trade-off between development rate and reproductive capacity. In most years (although not 2013 of this experiment), nearly all plants die owing to drought at approximately the same time, but *survival to flowering* differs greatly owing to varying rates of maturation [28,74]. The current study shows clear evidence of a viability trade-off with male reproductive success, with male selection for minor alleles in 2013 likely mediated through positive effects on flower size in this year of favorable growth conditions. Furthermore, consistency between 2013 and 2014 in the direction of allelic effects on male fitness suggests that such tradeoffs are intrinsic and contribute to the maintenance of big/slow alleles at minor frequencies within IM [18,27]. This is yet another of a growing body of examples relating antagonistic pleiotropy to polymorphism across diverse systems, e.g. [75].

Regarding prediction, selection on both quantitative traits and specific genetic loci with major effects can be quite strong [1,2,76]. However, both conceptual and logistical difficulties have separated phenotype-level and locus-specific approaches, limiting inference about the extent, nature, and magnitude of selection on genetic variants across the genome. Our results suggest that genotypic fitness is broadly estimable, and that these estimates can predict allele frequency change across generations (Fig 2). Unfortunately, it is much more difficult to determine the extent that apparent deviations between observed and predicted are due to sampling error as opposed to model error. The regressions of observed onto predicted Δ*p* for Paired contrasts (Fig 2B and 2D) are the simplest parametric relationships to interpret. The slopes for these, 0.61 and 0.62, suggest that response is less than predicted, but this conclusion is very tentative. Simple estimation error in the predictor of a linear regression causes a downward bias in the slope (here relative to one), even when there is no ascertainment bias [77]. This is non-trivial given that our SNP-specific predictions (and observations) of allele frequency change are encumbered with substantial estimation error [51].

Several biological factors may have reduced model accuracy. For example, we assumed that (a) there was no differential germination in the greenhouse (affected by genotype) when we grew progeny from maternal plants of 2013, (b) no seed bank contributed to the 2014 generation, and (c) no immigrant pollen or seed contributed to the 2014 population. Germination rates routinely differ between plant genotypes in an environment-dependent fashion, e.g. [78,79]. The field environment of 2014 (where plants germinated to produce our observed Δ*p*) is certainly different from the greenhouse (the offspring genotypes used to estimate *p*_*M*_ in 2013). This could cause substantial deviations between observed and predicted Δ*p*, although they would be limited to genomic regions containing “germination genes.” In contrast, many loci would be affected by violations of the assumptions regarding the seed bank and gene flow. If selection varies substantially among years, and all evidence indicates that IM experiences strong fluctuations [18, 23–26,28], a seed bank can moderate temporal changes in allele frequency [80]. *M*. *guttatus* does not have seed dormancy [81], and at present, we have no evidence that a seed bank exists for IM. If it does however, recruitment from the seed bank would probably act to reduce the magnitude of observed Δ*p* relative to predicted Δ*p*. Finally, there certainly is some level of gene flow into IM from other populations [54]. However, the fact that IM is a very large population [54], coupled with the observation of substantial allele frequency divergence from neighboring population [82], suggest that the rate of immigration is quite low (<< 1%). This level of gene flow might fundamentally alter long-term evolutionary dynamics (e.g. by introducing novel alleles), but should not have a dramatic effect on single-generation Δ*p* values.

A shortcoming of the Selection Component Analyses is that they do not provide an ecological explanation for the observed selection on SNPs. As in quantitative genetics, we can obtain such an understanding by replicating the measurement of selection across different populations (or the same population through time) and then correlating selection estimates with environmental or ecological variables. Mechanistic insights may also come from combining phenotypic measurements with genotyping and fitness assays, linking GWA with selection component analyses. In summary, a broader application of genomic selection component methods, coupled with environmental/phenotypic data and population monitoring through time, should help to resolve the limits of population genetic prediction.

## Materials and methods

### A. Field sampling and progeny testing

*Mimulus guttatus* (syn *Erythranthe guttata*) is a wild flower species (Family: Phrymaceae) abundant throughout western North America [83]. The IM population, located in the central Oregon cascades (44.402217 N, -122.153317 W, Elevation ~1400 meters), is described in detail elsewhere [23,25,28]. In 2013, whole plants distributed in a grid across the IM population were collected (at senescence) into coin envelopes. In 2014, we established three primary transects (each ~10m) horizontally across the face of the slope, with approximately equal vertical spacing between transects. The transects were further subdivided into perpendicular sub-transects which extended 0.3m on either side of the primary transect and were evenly spaced in 0.3m increments along the primary transect. We sampled five plants along each sub-transect by selecting the most proximal individual to a points placed at 10cm intervals. On July 15, 2014, we surveyed each transect and identified plants that would not progress to flower based on state of development relative to others in population. Assuming these plants would not have sufficient time to flower and set seed prior to season ending drought, this cohort (L) estimates *p*_*L*_ in Fig 1. To ensure sufficient DNA from L plants, we transplanted these individuals into moistened peat pots filled with potting soil and reared them to sufficient size for DNA extraction. We first sampled plants for the adult cohort of 2014 (*p*_*A*_ in Fig 1) on July 21, 2014. We only sampled adults once all plants within their sub-transect fully dried down. We collected whole plants, after confirming they had begun setting seed, into envelopes, so that both seed and maternal tissue could be separated for planting and DNA extraction, respectively. The remaining adults were harvested on July 27. Given seed collections from both years, we germinated and grew 2–4 progeny from each field plant in the University of Kansas greenhouse. We harvested dried leaf and calyx tissue from field collected parental plants and young leaves from greenhouse germinated progeny for subsequent DNA extraction [84]. To determine the overall proportion of the population that survived to flower in 2014, we surveyed a random set of 1000 seedlings marked early in the season at the nearby BR location [82]. Seven hundred of these plants eventually flowered.

### B. Library preparation, sequencing, SNP calling, and scoring read pairs

We collected paired-end sequence reads from 1936 experimental plants (2013: 207 field plants and 685 progeny; 2014: 383 field plants and 661 progeny) using Illumina technology. For field plants and their progeny, we generated genomic libraries using Multiplexed-Shotgun-Genotyping (MSG)[42], a form of RADseq [85] that uses a restriction enzyme to reduce genomic representation to homologous loci that are flanked by restriction cut sites. We digested genomic DNA from each plant using the frequent-cutting restriction enzyme MseI (NEB Biolabs). Each DNA sample was ligated to one of 96 distinct barcoded adaptors, each containing a unique 6 bp barcode. Each set of these barcoded samples is then pooled independently to create a sub-library. After PCR, we size-selected our library for 250-300bp fragments using a Pippin Prep (http://www.sagescience.com/products/pippin-prep/). We then performed PCR reactions at 12 cycles using Phusion High-Fidelity PCR Master Mix (NEB Biolabs) and primers that bind to common regions in the adaptors. In the PCR step, each sub-library was combined with one of 24 distinct Illumina indices allowing multi-plexing of the sub-libraries. To remove primer dimers, we did two rounds of AMPure XP bead cleanup (Beckman Coulter, Inc) using a 0.8 bead volume to sample ratio. Samples from different cohorts within each year (e.g. adults versus plants that failed to flower in 2014) were interspersed in library construction. However, the 2013 and 2014 plants were contained in different libraries and sequenced separately. Multiple sequencing runs were performed on the libraries from each year. Libraries were sequenced with 100-bp paired-end reads on the Illumina HiSeq 2500 with a 10% phiX spike-in. The program commands used to call SNPs in the MSG data are described in S1A Appendix. We suppressed Indels and all SNPs with more than two nucleotides segregating.

Sequencing and variant calling on the 187 reference panel genomes from IM was described previously [18]. We first imputed the few missing calls in these genomes and then extracted the sequence for each reference genome within each gene set (detailed procedures in S1B Appendix). We established gene sets as units for analysis. A set is either a single gene or a collection of closely linked (within 100bp) and/or overlapping genes. After suppressing genes prone to paralogous or otherwise spurious read mapping, 15,360 gene sets were retained for subsequent analysis (S1 Table). Finally, we noted that some SNPs were completely redundant–owing to perfect association in the reference panel, they always produced the same genotype likelihoods in field plants. We thinned sets of fully redundant SNPs to a single representative SNP leaving 1,523,410 SNPs for selection estimation.

The data units for likelihood calculations (Eq 1) are read-pairs scored for each polymorphic SNP that they overlap within a gene set. We aligned the read-pairs from each plant to the whole genome sequences, and within each gene set, and calculated *U*_[*plantID*],*i*,*j*_ for each possible genic-genotype [i,j]. *U*_[*plantID*],*i*,*j*_ is the likelihood for the full collection of read-pairs from a plant given that its diploid genic-genotype is [*i*,*j*], where *i* and *j* index genic haplotypes. Based on the low mismatch rate to genic haplotypes (as a whole), we set *ϵ* = 0.005 for calculation of Eq (1) described below. We calculated *U*_[*plantID*],*i*,*j*_ for each combination of gene set, plant, and genic-genotype using python scripts p1.py, p2.py, p3.py, p.Uij.2013.py and p.Uij.2014.py (S1 File).

### C. Drosophila melanogaster analysis

The *Drosophila* Synthetic Population Resource (DSPR) consists of two multi-parental, advanced generation intercross mapping populations [44,55]. Each population (A and B) was initiated with eight inbred founder strains, with one strain common to both populations (i.e., 15 founders in total). Following 50 generations of free recombination, a series of Recombinant Inbred Lines (RILs) were initiated by 25 generations of sibling mating. The founder genomes were sequenced to 50X coverage and the RILs subjected to RAD-seq using SgrAI, an 8-cutter, as the restriction enzyme [44,86]. Given these data, we are able to infer the mosaic founder haplotype structure of each RIL at >10,000 positions covering the genome.

We collected MSG RADseq data using the same protocol as described above for the Mimulus experiment, except that these data are 94bp single end sequences instead of the PE100 sequencing for Mimulus. We chose 60 of the RILs for the present study equally split between set A and set B of the DSPR. For each collection, there are only 8 possible ancestral genomes, but we ran the analysis blind to this information (thus inference among 15 possible ancestral alleles was required). The reads were processed with fastp (https://github.com/OpenGene/fastp) and then we mapped to the FlyBase r5.56 genome build (https://flybase.org/) and called SNPs following the procedures used for Mimulus (S1A Appendix). We used the annotation (dmel-all-r5.56.gff) to establish a list of 13,384 gene sets applying the same rules as for Mimulus (S3 Table). Next, we determined the intersection between SNPs within the ancestral genomes (final_snptable_foundersonly.txt downloaded from http://wfitch.bio.uci.edu/~dspr/) and those called in the MSG RIL data, a total of 107,878 bi-allelic SNPs (S4 Table). We found that 8900 of these 13,384 gene sets had at least one SNP scored in MSG data and could thus be used for downstream analysis. After eliminating uninformative reads, a total of 15,488,651 remained across the 60 RILs. We next adapted the Mimulus programs (python scripts p1.py, p2.py and p3.py in S1 File) to determine predicted ancestry based of the DSPR RILs and matched the inferred ancestry to the “known” ancestry of each RIL. The latter was established previously: We downloaded files HMMregA_R2.txt and HMMregB_R2.txt from http://wfitch.bio.uci.edu/~dspr/ (also available at https://datadryad.org/stash/dataset/doi:10.5061/dryad.r5v40). We processed the *D*. *melanogaster* reference into ‘gene units’ by the same method applied to the Mimulus genome. Read mapping and SNP calling were executed using the same techniques. The great majority of *D*. *melanogaster* reads overlap 3 or fewer SNPs and are thus less informative than the Mimulus read-pairs (S1 Fig). We then applied the inference programs using the 15 ancestral sequences of the DSPR as genic haplotypes.

### D. Likelihood of the field data with and without selection

Selection component analyses (SCA [43,47]) are based on population genetic models that predict allele frequency change from observations of viability, fecundity, and mating success [48]. SCA estimate selection from differences in allele frequency between distinct “cohorts” within a population, e.g. individuals that survive to reproduce and those that do not (viability selection) or those that acquire mates and those that do not (sexual selection) [49]. Given random sampling of individuals, the likelihood of the entire dataset (*L*) is a product across families:

$$L=\prod_{y=1}^{F}L_{y}$$ (2)

where *F* is the number of families and *L*_*y*_ is the likelihood for family *y*. Families consist of a single individual if that plant failed to survive to reproduce. For survivors, the family is the field plant and a sample of their progeny. The log-transformed likelihood:

$$Ln\;L=\sum_{y=1}^{F}Ln\{\sum_{i,j\geq i}^{K}P[M_{y}=i,j]P[{Data}_{y}|M_{y}=i,j]\}$$ (3)

where *P*[*M*_*y*_ = *i*,*j*] is the (prior) probability that the maternal genic-genotype has genic-haplotypes *i* and *j*. *K* is the number of distinct sequences for this gene set. *P*[*Data*_*y*_|*M*_*y*_ = *i*,*j*] is the probability of all data from family *y* (genetic and fitness measurements) given maternal genotype [*i*,*j*]. The family likelihood is:

$$P[{Data}_{y}|M_{y}=i,j]=U_{y,i,j}\prod_{z}^{O_{y}}V_{yz,i,j}$$ (4)

*U*_*y*,*i*,*j*_ is the probability maternal plant *y* produced the observed read-pairs given genic-genotype [*i*,*j*], *V*_*yz*,*i*,*j*_ is the probability of the observed read-pairs for offspring *z* of maternal plant *y* with genic-genotype [*i*,*j*], and *O*_*y*_ is number of genotyped offspring of maternal plant *y*. For individuals that fail to reproduce, [*Data*_*y*_|*M*_*y*_ = *i*,*j*] = *U*_*y*,*i*,*j*_. The likelihood for each offspring, *V*_*yz*,*i*,*j*_ in Eq 4, depends on whether that offspring is outcrossed or selfed (see Materials and methods section E). If offspring *yz* is selfed:

$$V_{yz,i,j}=\frac{1}{4}U_{yz,i,i}+\frac{1}{2}U_{yz,i,j}+\frac{1}{4}U_{yz,j,j}$$ (5)

We assume that each outcrossed progeny is sired independently and that

$$V_{yz,i,j}=\sum_{k=1}^{K}P\left[D_{yz}=k\right]\frac{1}{2}(U_{yz,i,k}+U_{yz,j,k})$$ (6)

*U*_*yz*,*v*,*w*_ is the probability of the observed read-pairs from offspring *yz* given that it has genic-genotype [v,w]. *P*[*D*_*yz*_ = *k*] is the probability that the sire of offspring *yz* transmitted genic-haplotype k to this offspring. The (1/2) reflects the equal probability of transmission for either maternal allele (*i* or *j*) to the offspring. Through all these calculations, we assume that recombination within gene sets has a negligible effect on the probabilities.

The various models of selection (Fig 1) are specified by different constraints on the genotype probabilities. Given the large number of genic-genotypes, the potential parameter space is very large. Here, we simplify by classifying all genic-haplotypes into two groups based on their allele at a particular SNP. We assume the sequences in a group are equivalent in terms of fitness effects. This reduces all genic-haplotypes at a gene set into two “alleles” for selection tests. This classification naturally changes with SNP chosen and thus we apply the procedure to each SNP in sequence. This simplification is a sensible first step, but we acknowledge that it may fail to capture the genotype-to-fitness mapping for many genes. In some cases, alternative alleles may be defined by numerous SNPs or indels within a gene [87,88] and fitness effects would be more naturally described with an allelic series. Our ‘binning’ of functionally distinct alleles could elevate the Type I error rate (we fail to see selection when it is occurring).

Let *S*_*R*_ represent the set of genic haplotypes that have the reference base at the focal SNP and *S*_*A*_ is the set with the alternative base. Then Eq (6) can be written:

$$V_{yz,i,j}=\sum_{v\in S_{R}}P\left[D_{yz}=v\right]\frac{1}{2}(U_{yz,i,v}+U_{yz,j,v})+\sum_{w\in S_{A}}P\left[D_{yz}=w\right]\frac{1}{2}(U_{yz,i,w}+U_{yz,j,w})$$ (7)

The frequency of the reference base (for the focal SNP) within the population of genic-haplotypes, *p*, is just $\sum_{k=1}^{K}\delta_{k}Q_{k}$, where *Q*_*k*_ is the frequency of haplotype k among the lines and *δ*_*k*_ is an indicator variable (1 if haplotype *k* carries the reference base and 0 otherwise). Of course, the frequency of the reference base can differ between the sequence line set and the natural population, and also between subsets of the natural population (e.g. alive versus dead). Let p* denote the frequency of the reference base in a specific field cohort, say adults in 2013 or zygotes in 2014. We adjust genic-haplotypes proportionally as a function of p*:

$$Q_{k}^{*}=Q_{k}\frac{p^{*}}{p}\;if\;k\in S_{R},Q_{k}^{*}=Q_{k}\frac{{(1-p}^{*})}{\left(1-p\right)}\;if\;k\in S_{A}$$ (8)

This is essentially a uniform inflation or deflation of haplotype frequencies based on the focal SNP. It allows us to write the likelihood equations explicitly in terms of allele frequencies at one SNP (e.g. *p*_*L*_, *p*_*A*_, and *p*_*M*_ in Fig 1) while retaining the full information from gene sets. For example, *P*[*M*_*y*_ = *i*,*j*], in Eq (3) becomes ${2Q}_{i}^{*}Q_{j}^{*}$ if i≠j or ${Q_{i}^{*}}^{2}$ if i = j. This is a function of known fixed values (p, *Q*_*i*_, *Q*_*j*_) and the parameter to be estimated (e.g. *p*_*A*_ if the maternal plant survived, *p*_*L*_ if not). Eq (7) becomes:

$$V_{yz,i,j}=\sum_{v\in S_{R}}Q_{v}\frac{p_{M}}{p}\frac{1}{2}(U_{yz,i,v}+U_{yz,j,v})+\sum_{w\in S_{A}}Q_{w}\frac{1-p_{M}}{1-p}\frac{1}{2}(U_{yz,i,w}+U_{yz,j,w})=p_{M}T_{1}+{(1-p}_{M})T_{2}$$ (9)

*T*_1_ and *T*_2_ distill all quantities in Eq (9) that are coefficients for *p*_*M*_ and (1−*p*_*M*_). The fact that these coefficients are determined entirely by the read-pairs from field plants and the set of genic-haplotypes means that they do not change with *p*_*M*_. Thus, the numerically intensive sum of Eq (6) need only be calculated once at the onset of a maximum likelihood search. We use Powell’s algorithm [89] to maximize likelihoods. At each SNP, we fit a series of models of increasing complexity (Fig 1). Likelihood ratio tests are used to evaluate whether more general models are superior to simpler models. The code to perform these tests was written in the C programming language, is described in S1C Appendix, and is included in S1 File.

### E. Mating system estimation

The MSG data (without the reference sequences) was used to determine individual offspring as outcrossed or selfed using BORICE [90]. The most informative SNPs for mating system estimation exhibit high coverage across samples and intermediate allele frequency. From the full set of MSG samples called simultaneously, we chose one SNP per gene with the highest count for (heterozygotes+the less frequent homozygote) using python program p4.py (S1 File). We then extracted genotype likelihoods for these SNPs directly from the vcf file and organized the samples into families (maternal plants with offspring) to produce a BORICE-format input file using python program p5.py (S1 File). We next thinned the dataset to SNPs with at least 800 called plants (across both years) producing the input file used for estimation of mating system (S6 Table) consisting of 2773 SNPs, each in a distinct gene and well distributed across all 14 chromosomes. We conducted preliminary MCMC runs to determine parameter step sizes, burn-in duration, and chain length. After setting these (Control file and the specific BORICE code are in S1 File), we estimated posterior probabilities for each offspring as outcrossed/selfed and the inbreeding level of maternal plants by combining four independent chains.

Considering offspring with at least one read at 100 or more SNPs, 10.1% were determined to be selfed in 2013 (54 of 537) versus 9.4% in 2014 (48 of 508). The remaining offspring, where there was insufficient data for estimation, were set as outcrossed for the subsequent selection analyses. While this classification may be incorrect for a few individuals, error has a minimal effect on parameter estimates given the absence of genotypic data for these offspring. The observed rate of selfing (ca. 10%) matches results from prior mating system studies of the IM population [91]. The detailed results are reported in S7 Table.

### F. Predicted and observed allele frequency change

We contrast different selection estimates in the common currency of predicted allele frequency change, Δ*p*. Considering the change from adults to zygotes of the next generation, the predicted change due to male selection is Δ*p* = (*p*_*M*_−*p*_*A*_)/2. This equation assumes no differential female fecundity (associated with the SNP) and that all progeny are produced by outcrossing (diploid loci are half male and half female). In fact, we found that ca. 10% of our offspring were derived from selfing (see section **E**). This could (slightly) inflate predicted change relative to observed change (Fig 2). However, given that the inflation is uniform, it does not affect arguments about significance, allele frequency, or trade-offs. The predicted change owing to viability selection in 2014 is calculated from model H_3_ (Fig 1) estimates, *p*_*A*_ and *p*_*L*_. The relevant relationship is *p*_*Z*_ = *α p*_*A*_+(1−*α*) *p*_*L*_, where *p*_*Z*_ is allele frequency in zygotes (before selection) and *α* is the fraction of individuals that survive to reproduce. For our experiment, we estimate *α* = 0.7 (see above in section **A**). Rearranging the equation, the predicted change owing to viability selection is Δ*p* = 0.3(*p*_*A*_−*p*_*L*_). The observed Δ*p* estimates (Fig 2) require an estimate of allele frequency in zygotes (*p*_*Z*_) from 2014. This can be estimated in several ways given the four models applied to the 2014 data (H_0_-H_3_ in Fig 1), but *p* from H_0_ is a robust choice. This value is always intermediate to parameter estimates from models that are more elaborate.

## Supporting information

S1 Table The gene sets are located to the genome sequence and the number distinct genic-haplotypes per gene set is reported. (XLSX)

S2 Table The number of SNPs covered per read-pair in the Mimulus field plants. After discarding read-pairs that overlap no SNPs, slightly more than 99 million remained.
(XLSX)

S3 Table The collection of genes and gene sets for the Drosophila application: "Gene.coordinates.txt". (TXT)

S4 Table Variants used in Drosophila application: “SNPs.in.both.txt”. (TXT)

S5 Table The most significant SNP per gene is reported for *p*_*A*_/*p*_*M*_ in 2013, *p*_*A*_/*p*_*M*_ in 2014, viability selection in 2014, and the change test (2013 adults to 2014 zygotes). The chosen for each test are reported on a separate sheet. Statistics from all model fits are reported for each SNP.
(XLSX)

S6 Table The BORICE formatted input file for mating system estimation. (GZ)

S7 Table The estimated posterior probabilities that each offspring is outcrossed and for the Inbreeding History (IH) level of maternal plant is reported. (XLSX)

S8 Table The estimated linkage disequilibrium is reported for all pairwise contrasts of the 587 male selection SNPs from 2013. (GZ)

S9 Table The best SNPs per gene evaluated by strength of evidence from two tests for selection: (A) male selection (*p*_*A*_/*p*_*M*_) in 2013 versus male selection in 2014, (B) male selection in 2013 versus viability selection in 2014, and (C) male selection in 2014 versus viability in 2014.
(XLSX)

S1 Fig The number of SNPs per read (Blue = Drosophila) or read-pair (Orange = Mimulus) is reported as a histogram. (JPG)

S1 File The programs used to perform calculations as described in Materials and methods. (GZ)

S1 Appendix The detailed methods are described for processing of MSG data, delineating gene sets and SNPs, selection component models, whole-genome data simulation, contrasts between gSCA tests, molecular population genetic tests for selection, and the genetic variance in fitness with multiplicative selection. (DOCX)
