# Supplementary figures and images for: Predicting evolutionary change at the DNA level in a natural Mimulus population
Source: PLoS Genet. 2021 Jan 13;17(1):e1008945. doi: 10.1371/journal.pgen.1008945 (PMC7837469; doi:10.1371/journal.pgen.1008945)

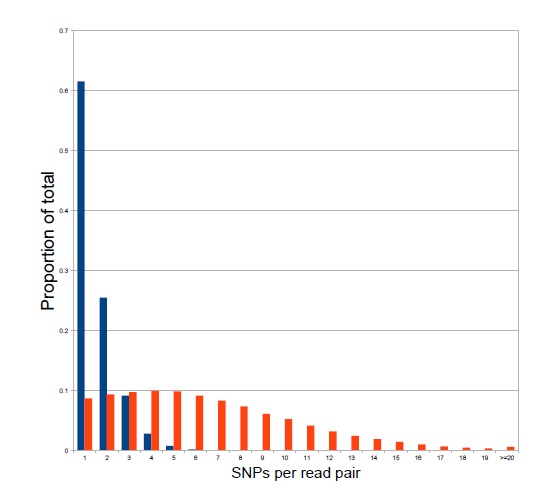

Supplement: S1 Fig — (JPG) [file pgen.1008945.s010.jpg]
