# Supplementary material for: Predicting evolutionary change at the DNA level in a natural Mimulus population
Source: PLoS Genet. 2021 Jan 13;17(1):e1008945. doi: 10.1371/journal.pgen.1008945 (PMC7837469; doi:10.1371/journal.pgen.1008945)
Supplement: S1 File — (GZ) [file pgen.1008945.s011.tar.gz › S1.File/Supplemental File 1 key.docx]

Supplemental File 1 key

Programs 1-3 score polymorphic sites within each read pair of each MSG plant and assign read pairs to gene sets.

1. p1.py

Input: sam file from each field plant, SNP list

Output: "hap1."+plant+".txt"

2. p2.py

Input: "hap1."+plant+".txt"

Output: "hap2."+plant+".txt"

3. p3.py

Input: "hap2."+plant+".txt"

Output: plant+“.genic.txt”

Programs 4 align read-pairs of MSG plants to reference line sequence to determine the likelihoods.

4. p.Uij.2013.py and p.Uij.2014.py

Input: plant+“.genic.txt” from all plants, sequences from all reference lines for gene set

Output: geneID+".linepairLL.txt"

An example of this output is provided from the 2013 plants for the gene I00422 (I00422.linepairLL.2.txt.gz). The format is: Field plant ID, reference line ID 1, reference line ID 2, LnL for read-pairs given the specified reference line sequences

Programs that perform maximum likelihood model fits to field data.

5. gsc.2013.c

Input: geneID+".linepairLL.txt", sequences from reference panel, key to family structure.

Output: Maximum likelihood fits (parameters and ML value) for model 0 and model 1 for each SNP in 2013 data.

6. gsc.2014.c

Input: geneID+".linepairLL.txt", sequences from reference panel, key to family structure.

Output: Maximum likelihood fits (parameters and ML value) for model 0-3 for each SNP in 2014 data.

7. gsc.13.14.c

Input: geneID+".linepairLL.txt", sequences from reference panel, key to family structure.

Output: Maximum likelihood fits (parameters and ML value) for models that do or not allow change in allele frequency from 2013 adults to 2014 zygotes.

8. gsc.2013.split.c

Input: geneID+".linepairLL.txt", sequences from reference panel, key to family structure.

Output: Maximum likelihood fits (parameters and ML value) for model 0 and model 1 for each SNP in 2013 data with the data partitioned into distinct subsets.

9. gsc.13.14.split.c

Input: geneID+".linepairLL.txt", sequences from reference panel, key to family structure.

Output: Maximum likelihood fits (parameters and ML value) for models that do or not allow change in allele frequency from 2013 adults to 2014 zygotes, with the data partitioned into distinct subsets.

The input files "out.self.2013.txt" and "out.self.2014.txt" specify each offspring as outcrossed or selfed.

Programs that prepare data for input to BORICE:

10. p4.py

Input: vcf containing all SNP calls from field plants for a specific chromosome

Output: Report on statistics for most informative SNP in each gene set ("best.borice."+chomosome+".txt")

11. p5.py

Input: vcf containing all SNP calls from field plants for a specific chromosome and "best.borice."+chomosome+".txt"

Output: BORICE formatted datafile with genotype likelihoods reported for each individual and SNP.

12. Borice.Genomic.dt1.py

Input: Borice formatted SNP data. Program settings used for the Borice run are read in from “Control.txt”

Output: Mating system estimation files

Programs for whole genome simulation without selection:

13. linepair.LL.fullsim.2013.py

14. linepair.LL.fullsim.2014.py

Program to calculate molecular test statistics within windows around select nucleotide positions:

15. molecular.tests.py

Program to calculate LD statistics among select nucleotide positions:

16. LD.among.selected.loci.py
