# Supplementary material for: Predicting evolutionary change at the DNA level in a natural Mimulus population
Source: PLoS Genet. 2021 Jan 13;17(1):e1008945. doi: 10.1371/journal.pgen.1008945 (PMC7837469; doi:10.1371/journal.pgen.1008945)
Supplement: S1 Appendix — (DOCX) [file pgen.1008945.s012.docx]

Supplemental Appendix

| Appendix | Page |
| --- | --- |
| A. Bioinformatic processing of MSG data | 2 |
| B. Delineating gene sets and SNPs | 3 |
| C. Selection component models | 4 |
| D. Whole-genome data simulation | 5 |
| E. Contrasts between gSCA tests | 6 |
| F. Molecular population genetic tests for selection | 7 |
| G. The genetic variance in fitness with multiplicative selection | 10 |

A. Bioinformatic processing of MSG data.

We de-multiplexed the fastq files from the sequencing into sample specific sequence files. We processed read pairs with Scythe (https://github.com/vsbuffalo/scythe/) to remove adaptor contamination and Sickle (https://github.com/najoshi/sickle/) to trim low quality sequence. Using BWA with default parameter values [1], we mapped the processed reads to the v2 draft of the *Mimulus guttatus* genome (http://www.phytozome.net/). The read mapping for plant X was executed using:

bwa mem [reference genome] ss.X.[read1].fq ss.X.[read2].fq | samtools view -bS - | samtools sort - -o X.sorted.bam

Here ss.[read1].fq ss.[read2].fq are the Scythe and Sickle process read pair files for this plant. We next used picard tools to add read groups to the sorted bam files:

java -jar AddOrReplaceReadGroups.jar I= X.sorted.bam O=RG/ X.sorted.bam SO=coordinate RGID=SeqRUN# RGLB= X.sorted.bam RGPL=illumina RGPU= X.sorted.bam RGSM= X.sorted.bam VALIDATION_STRINGENCY=LENIENT

Next we indexed these bams files using:

samtools index RG/X.sorted.bam

Given sorted bam files, we called SNPs with the UnifiedGenotyper algorithm in the Genome Analysis ToolKit [2] (GATK) using all MSG data simultaneously:

java -jar GenomeAnalysisTK.jar -R /home/jkk/Mguttatus_256_v2.0.fa -T UnifiedGenotyper -glm BOTH -L [1mb chromosomal section] -I [all bams from both years] - ploidy 2 -rf BadCigar -o [1mb chromosomal section].vcf

Finally, we concatenated the VCF files across all chromosomal segments.

B. Delineating gene sets and determining SNPs across sequenced lines and MSG samples

We analyzed the reference panel sequences to identify regions with aberrant features; regions likely misassembled in reference genome. We suppressed genes if the mean read depth (across the 187 IM lines) was lower than the 10th or higher than the 90th percentile of the distribution. These thresholds were based on observations from an RNAseq experiment [3] indicating spurious transcript mapping to genes in both the low and high portions of the read depth distribution. After thinning, we combined genes into a common set if they had overlapping reading frames or the distance between start/end was less than 100bp. The resulting list (S1 Table) contained 15,901 genes (e.g. N03296) or gene combinations (e.g. N03297.N03298). Finally, we excluded genes if they lacked RADtags (field plants provided no data) or lacked polymorphism (no SNP alleles at greater than 5% in the field samples). The final set included 15,360 gene sets analyzed for selection.

To create complete genic sequences for each of the 187 lines of the reference panel, we imputed bases called as N in our previous analysis [4]. For each polymorphic site, we determined the one other SNP in the gene exhibiting the highest linkage disequilibrium (measured as r^2^) with the focal SNP. Given the base observed at this “partner SNP”, we calculated the conditional probability of reference and alternative base at the focal site. We could then probabilistically impute the unobserved base at a SNP initially reported as N given the observed base at the partner SNP. Given the complete line sequences within each gene set, we determined the number of distinct sequences for input to the selection programs (described below in section C).

SNP calls were generally congruent between the whole genome sequences and the MSG data. Polymorphisms in the MSG data were generally observed at the same positions (genome coordinates) as in the reference panel genomes, with the same bases segregating in each dataset. There are more polymorphisms in the reference panel because (a) much of the genic sequence is not contained within RADtags, and (b) rare alleles in the reference lines were not always ascertained in the MSG data even if predicted to segregate within RADtags. The initial set consisted of 2,854,081 SNPs. However, we eliminated SNPs if the minor allele was less than 5%. Allele frequency was determined as the average of model 0 estimates for p from 2013 and 2014, respectively (model 0 is no selection). We next noted SNPs that produce exactly equivalent genotype inferences in the field plants, typically SNPs that are perfectly associated in the reference panel (r^2^ = 1). We randomly selected one per set for testing given that all perfectly associated SNPs yield the same test outcomes. After these filters, 1,523,410 remained for selection component estimation.

Given the reference panel sequences for a gene set, we determined the likelihood that the collection of read-pairs from each plant given each possible genic-genotype ($U_{\left[ plantID \right],i,j}$ in eq (1) of main paper). This first required that we extract the SNP genotypes for each read pair and organize these into sets mapping to each gene set (programs p1.py, p2.py and p3.py in S1File). The alignment of read-pairs to each possible genic-genotype is executed by the programs p.Uij.2013.py (for plants from 2013) and p.Uij.2014.py (plants from 2014). The likelihood is determined by the extent of sequence mis-match between read-pairs and genic-genotype [i,j], and the mismatch probability, ϵ.

C. Selection component models

The maximum likelihood fits to all models in Figure 1 were performed using gsc.2013.c (for 2013 tests), gsc.2014.c (for 2014 tests), and gsc.13.14.c (for the cross generation change test). For the designated gene set, these programs first read in the $U_{\left[ plantID \right],i,j}$ values for each individual in the relevant field year (or years). These are used to calculate $T_{1}$ and $T_{2}$ of eq. (9) and stored for subsequent calculations. The calculation of $T_{1}$ and $T_{2}$ accounts for the classification of offspring as outcrossed or selfed (information contained in "out.self.2013.txt" and "out.self.2014.txt", respectively). The 2013 program then fits two models in sequence. Model 0 involves a single parameter, p, to describe allele frequency in maternal plants and their offspring. Bounded optimization using the Powell algorithm is applied with code slightly modified from [5]. Model 1 involves two parameters, $p_{A}$ and $p_{M}$, the optimization is initiated for the ML estimate from p from model 0. Equations 2-9 are applied in each call to the likelihood function as the algorithm tests different parameter values.

The 2014 program (gsc.2014.c) has the same structure but with four models fit in sequence (Fig 1). The first (model 0) has a single allele frequency for each cohort (plants that failed to flower, reproductive adults, and successful males). Model 1 allows $p_{M}$ to differ from the other cohorts (with $p_{A}=p_{L}$). Model 2 allows viability selection ($p_{L}$ can differ from $p_{A}$), but not male selection (with $p_{A}=p_{M}$). Finally, model 3 allows $p_{A}\neq p_{L}\neq p_{M}$. The parameter search for each model is initiated using estimates from simpler models.

The “change test” is performed on data from both field populations considered jointly. The code (gsc.13.14.c) includes the read statements from both previous programs for $U_{\left[ plantID \right],i,j}$ values, and performs parallel calculations of the $T_{1}$ and $T_{2}$ statistics for each year. The first model involves a two parameters $p_{A}$and $p_{M}$ describing adults and males in 2013. The likelihood for the 2014 component of the data is obtained by applying model 0 to that year using $p_{A}(2013)$ for allele frequency. The second model has three parameters, the additional quantity is a single distinct allele frequency for the 2014 data. ML are obtained using the Powell algorithm as previously. The final two programs, gsc.2013.split.c and gsc.13.14.split.c, perform the cross validation tests (Figure 5 of main paper). Each performs two sets of model fits per SNP, on the even and odd numbered families respectively. They are otherwise equivalent to the programs performing the same tests on the full dataset, gsc.2013.c and gsc.13.14.c, respectively. All *.c programs are contained in S1 File.

D. Whole-genome data simulation

We simulate data starting from the reference panel sequences for each gene set. The data for a maternal field plant is created by randomly selecting alleles from the genic-haplotype set. The probability that a particular haplotype is selected is proportional to its frequency in the reference panel. Given two alleles, we simulate read-pairs as copies from these two alleles. The number of read-pairs, and their locations within genes, is copied directly from the real data for that plant. As a consequence, the size of the simulated dataset exactly matches the real dataset. We simulate offspring by randomly sample male haplotypes from the reference panel, if the offspring is indicated to be outcrossed (again matching this classification in the real data). Given maternal genic-genotypes and paternal genic-haplotype, the offspring data is simulated according the number/location of read-pairs in each offspring. For selfed progeny, the offspring genic-genotype is determined by Mendelian segregation of the two maternal alleles. We assume no error in transmission (nucleotides in the offspring alleles exactly match those in parental sequences, which perfectly match reference panel sequences). Because the sampling of haplotypes is random, this procedure generates data under the null model of no selection.

Simulated datasets were created using the programs “linepair.LL.fullsim.2013.py” and “linepair.LL.fullsim.2014.py” (S1 File). These programs progress through all families present in each year of data, simulating parents and offspring in sequence. Once the simulated data is created, these programs perform the matching of (simulated) read-pairs to each possible genic-genotype – essentially executing the calculations performed by the programs p.Uij.2013.py and p.Uij.2014.py on the real data. The outputs of the simulation at this stage are structurally equivalent to the $U_{\left[ plantID \right],i,j}$ calculations for the real data. As consequence, the simulated data can thus be input directly in the C programs (described above in section C) applied the real data. All the ‘design features’ are equivalent between simulated and real datasets, they differ only in the determination of SNP alleles within read-pairs.

We used this simulator to created replicate whole genome datasets under the condition of neutrality. These outputs were subject to the same post testing filters as the real data to evaluate ascertainment biases (see main text arguments on prediction, Fig 5). However, the consideration of LRT from all 1,523,410 tests performed on a simulated dataset (${p_{A}}/{p_{M}}$ for 2013 simulation, viability and ${p_{A}}/{p_{M}}$ for 2014) also provides a necessary evaluation of our testing procedures. The p-values from our tests are based on the asymptotic likelihood expectation: the LRT values should follow a chi-square-1 distribution if there is no selection. The simulations provide finite datasets (exactly the same size as the real data) under the null hypothesis. We find excellent agreement with chi-square-1 for the distribution of LRT values from all three tests on simulated data. The first four moments of the empirical distribution match expected very closely.

E. Contrasts between selection tests

Each of the 1,523,410 SNPs was subject to three tests for selection: ${p_{A}}/{p_{M}}$ in 2013, viability in 2014, and ${p_{A}}/{p_{M}}$ in 2014. A single SNP per gene is reported in Figures 2A,C, 4, and 5. In these cases, it was the most significant SNP within the gene for the specific test considered in that analysis (figure panel). We performed ascertainment of ‘best SNP per gene’ when considering pairs of tests. Here, we chose the SNP that that pair of tests, e.g. ${p_{A}}/{p_{M}}$ in 2013 versus viability in 2014, with the strongest evidence of effect across both tests. We combined the p-values from each test using Fisher’s combined probability statistic [6]:

$X^{2}=-2 Ln(p_{1})-2 Ln(p_{2})$

where $p_{1} and p_{2}$ are the p-values from each test of selection. If the tests are independent, then a combined p-value is obtained from the chi-squared distribution with 4 degrees of freedom. The tests between selection estimates from different years are based on wholly different measurements and are thus independent. The combined p-value is thus a valid means to combined results from (A) ${p_{A}}/{p_{M}}$ in 2013 versus ${p_{A}}/{p_{M}}$ in 2014 and (B) ${p_{A}}/{p_{M}}$ in 2013 versus viability in 2014. However, the tests within 2014 (${p_{A}}/{p_{M}}$ in 2013 versus viability in 2014) are not independent and the predicted Δp values from each test are negatively correlated (even under the null hypothesis of no selection). Here, we use the combined p-value only as an indicator for relative strength of evidence, and not as the probability of obtaining an $X^{2}$ as large as observed if the null hypothesis were true.

All pairwise contrasts between gSCA tests (15,360 genes) are given in S9 Table.

F. Molecular population genetic tests for selection

We analyzed the 187 genome sequences in our reference panel [4], prior to imputation of base calls for genic-haplotypes (section B above). We calculated S, Pi, Tajma’s D, and Z_focal_ within genomic windows, treating uncalled bases as missing data, using the program “molecular.tests.py” (contained in S1 File). S = the number of polymorphisms in the window, excluding the focal SNP. Pi = the average number of nucleotide differences between sequences in the window (excluding the focal SNP) divided by 200 (the number of bases). Tajma’s D was calculated using the formulas in [7] given Pi and S. Z_focal_ is the Z_ns_ [8] value specific to the contrast between the focal SNP and all other SNPs within the window. We used a window size of +/- 100 bp of the focal SNP for all analyses reported.

The input file to molecular.tests.py, IM.genomat.all.txt, is the genotype matrix from our GWA study using the reference panel [4]. This file contains 10,199,238 bi-allelic SNPs, the subset to pass filters for the GWA study. The filtering almost certainly eliminated some rare alleles from the genotype matrix, which is evident from the minor allele frequency spectrum for the SNPs included in IM.genomat.all.txt:

The deficiency of SNPs with a minor allele <2% is likely an ascertainment effect. Consequently, our estimated values for Pi and S are likely below the population values, and the estimated value for Tajima’s D is likely inflated. However, these biases are shared between the contrasted groups (windows around selected-SNPs and control SNPs).

We compared the means of windows from selected- and control-SNPs using ANOVA. The differences were hugely significant for 2013-male selection (Fig 5C). The contrasts for 2014 selection windows (selected-SNPs and control) are depicted below, with the same conventions as for Fig 5 in main text:

The direction of differences for 2014 windows are the same as for 2013, although not all tests are significant. ANOVA tables are given for both 2014 episodes below:

| Viability selection in 2014 | | | | | Male selection in 2014 | | | | |
| --- | --- | --- | --- | --- | --- | --- | --- | --- | --- |
| S |  |  |  |  | S |  |  |  |  |
| Source | DF | Adj MS | F-Value | P-Value | Source | DF | Adj MS | F-Value | P-Value |
| type | 1 | 418.28 | 12.31 | 0 | type | 1 | 355.96 | 10.96 | 0.001 |
| Error | 3559 | 33.98 |  |  | Error | 3939 | 32.47 |  |  |
| Total | 3560 |  |  |  | Total | 3940 |  |  |  |
|  |  |  |  |  |  |  |  |  |  |
| Pi |  |  |  |  | Pi |  |  |  |  |
| Source | DF | Adj MS | F-Value | P-Value | Source | DF | Adj MS | F-Value | P-Value |
| type | 1 | 49.448 | 13.69 | 0 | type | 1 | 38.105 | 11.45 | 0.001 |
| Error | 3559 | 3.612 |  |  | Error | 3939 | 3.327 |  |  |
| Total | 3560 |  |  |  | Total | 3940 |  |  |  |
|  |  |  |  |  |  |  |  |  |  |
| Tajima's D |  |  |  |  | Tajima's D |  |  |  |  |
| Source | DF | Adj MS | F-Value | P-Value | Source | DF | Adj MS | F-Value | P-Value |
| type | 1 | 4.056 | 2.77 | 0.096 | type | 1 | 3.313 | 2.25 | 0.134 |
| Error | 3500 | 1.466 |  |  | Error | 3878 | 1.472 |  |  |
| Total | 3501 |  |  |  | Total | 3879 |  |  |  |
|  |  |  |  |  |  |  |  |  |  |
| Z focal |  |  |  |  | Z focal |  |  |  |  |
| Source | DF | Adj MS | F-Value | P-Value | Source | DF | Adj MS | F-Value | P-Value |
| type | 1 | 0.2635 | 5.48 | 0.019 | type | 1 | 0.1307 | 2.54 | 0.111 |
| Error | 3500 | 0.0481 |  |  | Error | 3878 | 0.0514 |  |  |
| Total | 3501 |  |  |  | Total | 3879 |  |  |  |

G. The genetic variance in fitness with multiplicative selection

Consider a multiplicative model of selection where allele 0 is favored relative to allele 1. The diploid genotype fitnesses are: w00 = 1, w01 = (1-s), and w11= (1-s)^2^. If p is the frequency of the favored allele, then

$$\Delta p=\frac{(1-p)ps}{1-(1-p)s}$$

[9]. Setting p = 0.5 and $\Delta p=0.045$, we obtain s = $0.165$. Now consider 500 loci of this kind with fitness combining multiplicatively across loci. Letting *i* index loci, the mean fitness is then

$$\bar{w}=\prod_{i=1}^{500} \left( 1-\left( 1-p_{i} \right)s_{i} \right)\approx4.04 x{10}^{-38}$$

This implies that the fitness of the average individual is extremely small relative to the “perfect genotype” (homozygous for allele 0 at all loci). However, as was emphasized 50 years ago in the genetic load debate, e.g. [10], the perfect is never observed in a population. The variance in fitness among individuals residing in the population is a more meaningful calculation. The graph below depicts the distribution of relative fitness values (mean = 1) for 500 loci with s = $0.165$.

Noting the log-scale for relative fitness, we see that the multiplicative model predicts a very high variance in relative fitness (~3140) and a strongly right-skewed distribution. Here, the fitness of the top 1% of individuals is >680 times the fitness of the median individual in the population. This level of variability and skew may simply be too large relative to what is possible for natural populations with limits on reproductive capacity.

References

1. Li H, Durbin R. Fast and accurate short read alignment with Burrows-Wheeler Transform. Bioinformatics. 2009;25:1754-60.

2. McKenna A, Hanna M, Banks E, Sivachenko A, Cibulskis K, Kernytsky A, et al. The Genome Analysis Toolkit: A MapReduce framework for analyzing next-generation DNA sequencing data. Genome Research. 2010;20(9):1297-303. doi: 10.1101/gr.107524.110.

3. Colicchio JM, Monnahan PJ, Kelly JK, Hileman LC. Gene expression plasticity resulting from parental leaf damage in Mimulus guttatus. The New phytologist. 2015;205(2):894-906. Epub 2014/10/10. doi: 10.1111/nph.13081. PubMed PMID: 25297849.

4. Troth A, Puzey JR, Kim RS, Willis JH, Kelly JK. Selective trade-offs maintain alleles underpinning complex trait variation in plants. Science. 2018;361(6401):475-8. doi: 10.1126/science.aat5760.

5. Press WH, Teukolsky SA, Vetterling WT, Flannery BP. Numerical recipes in C. 2nd ed. Cambridge: Cambridge University Press; 1992.

6. Fisher R. Statistical Methods for Research Workers. 12 ed. Edinburgh: Oliver and Boyd; 1954.

7. Tajima F. Statistical method for testing the neutral mutation hypothesis by DNA polymorphism. Genetics. 1989;123:585-95.

8. Kelly JK. A test of neutrality based on interlocus associations. Genetics. 1997;146(3):1197-206.

9. Hartl DL, Clark AG. Principles of population genetics. Sunderland, Massachusetts: Sinauer associates; 1989.

10. Wallace B. Genetic Load. Its Biological and Conceptual Aspects. Englewood Cliffs, N.J.: Prentice-Hall; 1970.
